# Concrete Shrinkage Behavior Under Varying Degrees of Restraints Using DIC

**DOI:** 10.3390/ma19153220

**Published:** 2026-07-28

**Authors:** Haolin Guo, Yajie Zhang, Runze Du, Shengfa Fang, Shaowei Wu, Yihong Guo, Jianfu Lv

**Affiliations:** 1 College of Aerospace and Civil Engineering, Harbin Engineering University, Harbin 150001, China; guohaolin@hrbeu.edu.cn (H.G.); 18646037739@163.com (Y.Z.);; 2 Engineering Department State Grid (Shanghai), Smart Grid R&D Investment Co., Ltd., Shanghai 200126, China; 3 State Key Laboratory of Nuclear Power Safety Technology and Equipment, China Nuclear Power Engineering Co., Ltd., Shenzhen 518116, China

**Keywords:** varying degrees of restraint, DIC, circular ring test, shrinkage, concrete

## Abstract

**Highlights:**

- Investigated four restraint levels (0%, 35%, 55%, 75%) via inner steel ring thickness.
- Examined effect of graded restraint on concrete shrinkage behavior.
- Quantified shrinkage using digital image correlation and strain gauges.

**Abstract:**

Concrete shrinkage is significantly influenced by the restraint level, and cracking often occurs under specific restraint conditions, thereby adversely affecting structural performance. Investigating the effect of restraint on shrinkage cracking is of great significance for enhancing early-age durability and ensuring structural safety. In this study, four distinct restraint levels (0%, 35%, 55%, and 75%) were established by varying the thickness of the inner steel ring. The influence of varying degrees of restraint on the shrinkage behavior was investigated, with digital image correlation (DIC) and internal strain gauge measurement employed to observe the strain and predict the risk of cracking. As the degree of restraint increases, the inner steel ring inhibits the free radial shrinkage of concrete more significantly, thereby inducing greater tensile strains at both the outer circumferential surface and the interior. The surface strain accumulation far exceeds the interior response due to the drying gradient. During the first 60 h, the shrinkage strain measured by both methods exhibited the most rapid evolution, indicating a critical high-risk period for cracking. These results advance the understanding of restraint effects in concrete and comprehensively clarify the relationship between the degree of restraint and the shrinkage, which accurately captures the evolution of shrinkage, facilitates the transition from empirical to quantitative design for crack-resistant materials and supports their customized optimization under practical engineering loading conditions.

## 1. Introduction

Concrete cracking is primarily driven by the interaction between material shrinkage and structural restraint. Shrinkage refers to the inherent volumetric reduction in the cement matrix, which occurs via chemical, plastic, thermal, autogenous, and drying mechanisms [1]. In engineering practice, restraint typically originates from rigid structural connections, such as beam–column, wall–wall, and beam–slab joints. These connections restrict the natural shrinkage of early-age concrete, generating internal tensile stresses. Cracking ultimately initiates when these induced stresses exceed the tensile strength of the material [2].

In engineering practice, concrete cracking remains a persistent challenge. Extensive observations reveal that the near-surface region typically undergoes significantly greater shrinkage than the internal core. This differential is driven by moisture evaporation [3,4], surface cooling, and interfacial restraint gradients [4], which collectively accelerate early-age surface cracking and compromise both structural durability and interfacial bonding [4]. Beyond the macroscopic restraint imposed by the external boundaries (such as a steel ring setup), this non-uniform shrinkage induces internal self-restraining forces that further elevate tensile stresses [5]. As Bažant noted, the strains generated by non-uniform drying frequently exceed the tensile strain capacity of concrete, initiating strain softening and subsequent cracking [6]. Accurately characterizing the interplay between varying degrees of restraint and localized surface shrinkage is essential for advancing concrete modeling, design, and durability assessments.

To experimentally evaluate the cracking potential, the restraint ring test is widely adopted due to its operational simplicity and analytical tractability [7]. While both active and passive configurations exist, passive tests are generally favored for their convenience and broader applicability. The equipment of the passive test encompasses circular [8,9,10], elliptical [11,12,13], eccentric [14], and double-ring [15,16,17] geometries, with the circular ring test serving as the predominant standard for assessing restrained shrinkage. Conventionally, these tests rely on strain gauges affixed to the inner steel ring to indirectly quantify the deformation of concrete and identify the time of cracking. However, this traditional measurement approach presents significant limitations. Strain gauges are inherently prone to data drift—an anomalous decrease in measured strain over time even under unloaded conditions—rendering them unreliable for long-term monitoring. Furthermore, for this method, which infers concrete behavior indirectly through the steel mold, the accuracy of the measurement is highly sensitive to the material properties, thickness, and dimensions of the inner steel ring itself [18].

To overcome the inherent limitations of discrete internal sensors, digital image correlation (DIC) offers a robust, non-contact alternative for high-precision deformation monitoring [19]. Bypassing the need for physical instruments like displacement and strain gauges, DIC extracts full-field kinematic data entirely through image analysis. The technique works by tracking the grayscale distribution of target subsets in deformed images relative to a reference state, enabling the accurate quantification of surface displacements and strain fields within the area of interest [20,21]. Since it is fundamentally impractical to bond traditional strain gauges directly to the surface of early-age or fresh concrete, the non-contact nature of DIC offers a powerful approach for capturing macroscopic surface deformation. Accordingly, researchers have widely adopted the technique to capture the plastic shrinkage strain of cementitious materials [22,23,24,25,26,27] and to assess cracking mechanisms during the plastic stage [23,26]. DIC uniquely enables the continuous monitoring of crack initiation, propagation, and spatial distribution—key insights unavailable via discrete sensors. Furthermore, specialized DIC post-processing algorithms have been developed to quantify critical crack parameters, including width, length, and area [25]. This approach fundamentally enhances the analytical accuracy of shrinkage-induced cracking research, with the obtained measurements showing a strong correlation with optical microscopy verification.

Despite its proven efficacy in studying general cementitious materials, the direct implementation of DIC for strain measurement in structural concrete remains limited. Notably, Zhang et al. [18] successfully employed DIC in restrained ring tests to correlate early-age strain fields with crack evolution. Nevertheless, that study presents two critical research gaps: it did not account for the influence of variable steel ring restraint levels on concrete shrinkage performance, nor did it explore combining DIC with complementary testing methods to improve the accuracy of measured strain data.

While the traditional ring test is widely adopted, it predominantly relies on inner steel gauges, assuming a uniform stress distribution and failing to capture the complex strain dynamics on the exposed surface [22]. Furthermore, existing studies mostly evaluate cracking under a single, severe restraint condition [7]. At present, there is a critical research gap regarding how varying restraint degrees dynamically affect the differential shrinkage between internal structural resistance and local surface deformation. In this study, DIC is applied to investigate the effect of restraint levels on shrinkage strain in concrete rings. Accordingly, experiments were conducted with four predefined restraint levels: 0%, 35%, 55%, and 75%, using 100 mm thick concrete rings. DIC was used to monitor the shrinkage pattern of the top surface under restraint conditions for 180 days, while strain gauges were used to measure the shrinkage evolution of the inner steel ring during the initial 15 days. The influence of restraint levels on the shrinkage behavior of concrete with non-penetrating cracks was analyzed and summarized. For the first time, the influence of varying restraint levels on concrete early-age shrinkage is investigated, offering a reference for addressing early shrinkage cracking in engineering practice with diverse restraint scenarios. This approach circumvents the limitations of traditional, “one-size-fits-all” crack mitigation strategies, providing a quantitative scientific foundation for the precise tailoring of crack-resistant materials.

## 2. Raw Materials and Experimental Methods

### 2.1. Materials and Sample Preparation

This study adopts the mix proportions of concrete used in nuclear power plant facility construction. Based on on-site preliminary tests conducted in Harbin, the concrete grade is specified as C50. The mix shown in Table 1 consists of ordinary Portland cement, fly ash, fine aggregate, and coarse aggregate.

Both the cement and fly ash utilized in this study were procured from local manufacturers in Harbin, China. Their principal chemical compositions are detailed in Table 2.

**Figure 1:**
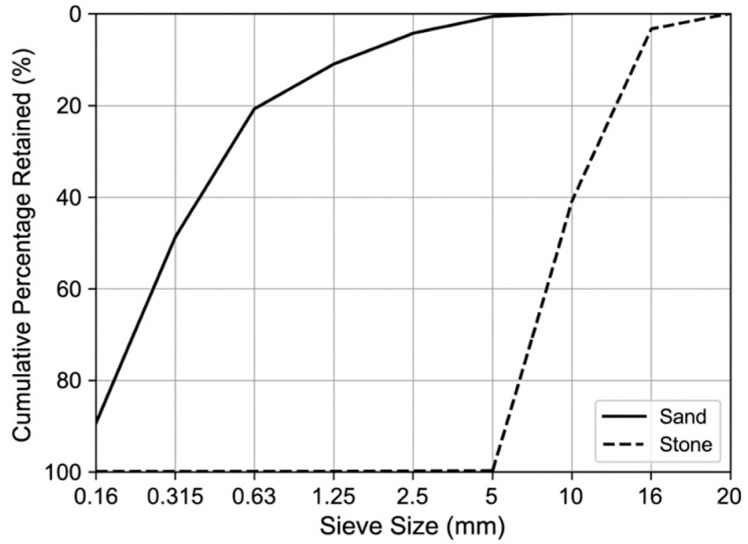
Particle size distribution curves of the fine and coarse aggregates.

The coarse aggregate (crushed stone) was sourced from the Acheng Xiaolingzi Quarry, located in Acheng District, Harbin City, Heilongjiang Province, China, while Songhua River sand was employed as the fine aggregate. Their particle size distribution (PSD) curves are plotted in Figure 1. The coarse aggregate was divided into two distinct size fractions: 5–10 mm and 10–20 mm. In accordance with the Chinese standard JGJ 52-2006, the apparent densities of the fine and coarse aggregates were determined to be 2.54 × 10^3^ kg/m^3^ and 2.70 × 10^3^ kg/m^3^, respectively.

**Figure 2:**
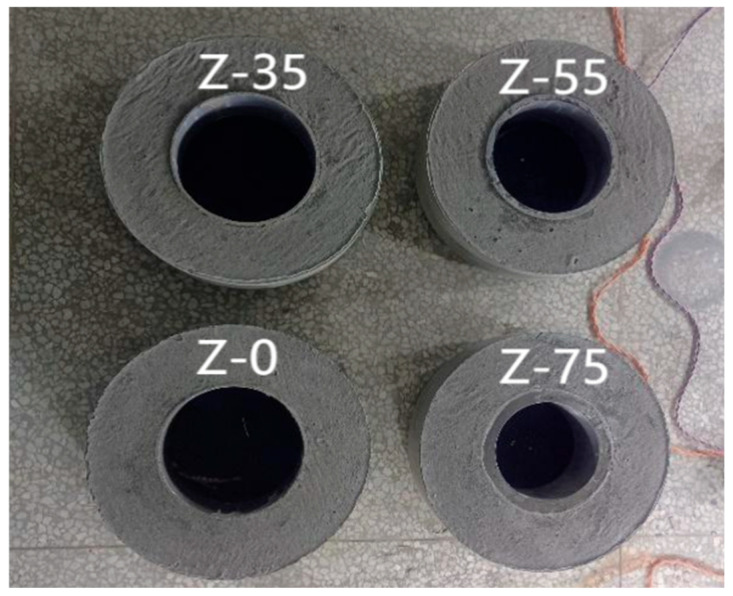
Varying degrees of restraints on concrete specimens.

Aggregates and cementitious materials were meticulously weighed and added sequentially into a horizontal concrete mixer. Following a one-minute period of mixing, the predetermined amount of water was added, and the mixture was left to stand for a further three to five minutes. After thorough mixing, fresh concrete was poured into the annular mold, and its surface was leveled to obtain a smooth and uniform finish. For each mix proportion, two replicate specimens were prepared for testing. The slump of fresh concrete was measured to be 110 mm. To apply the DIC method, speckle patterns are manually fabricated, or natural surface speckles are adopted for data acquisition. Speckle patterns enhance the surface features of specimens. During testing, the clarity of captured images is degraded by variations in speckle grayscale gradients, surface reflections, and lighting conditions. However, artificially sprayed speckle patterns often fail to adhere stably when the surface of concrete is moist or the concrete is in the early curing stage, and they also significantly perturb the moisture transport characteristics of the surface, thereby reducing the recognizability and stability of speckle textures in subsequent DIC analysis. To simultaneously address the challenges of speckle quality, moisture transport, and surface optical reflection, a novel surface treatment strategy was proposed and adopted in this study. After approximately 30–40 min of initial setting, the concrete surface was covered with a layer of breathable gauze to reduce surface reflection during hardening, optimize natural speckle quality, and further improve the measurement accuracy of DIC (as shown in Figure 2). Through careful optimization of the optical focus and illumination, the matching uncertainty of VIC-2D by utilizing the gauze-induced surface texture was successfully maintained below 0.01 pixels. This magnitude is well within the acceptable threshold, ensuring highly reliable DIC measurements. The gauze was removed approximately three hours after casting. For unconstrained specimens, the internal removable steel ring was also removed (seen in Figure 2). During the controlled experimental period, the indoor temperature and humidity conditions were maintained constant. Following the removal of gauze, restraint strain data were systematically gathered at one-minute intervals for 15 days using a strain-testing instrument. Images of shrinkage were recorded by an industrial camera every 10 min during the initial two days, and subsequently at three-hour intervals. The temporal intervals were selected with meticulous care to ensure the acquisition of precise and consistent strain data, while concurrently circumventing the necessity for excessive storage capacity.

Two replicates were tested for each restraint condition. For the strain gauge and virtual extensometer data, the reported values are averages of the two replicates. For full-field DIC contour maps, a single representative specimen is shown. While the small sample size (*n* = 2) precludes plotting error bars, parallel observations confirmed negligible variability and highly consistent deformation patterns between replicates.

### 2.2. Experimental Design and Methods

#### 2.2.1. Parameters of Circular Restraint Ring

**Figure 3:**
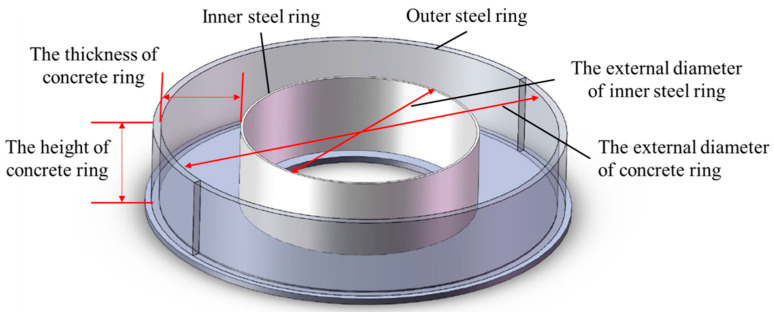
Circular restraint ring.

In the experiment, a circular restraint ring was utilized, comprising an interior steel ring with an external diameter of 225 mm and a concrete ring with an external diameter of 425 mm. The concrete ring exhibited uniform dimensions of 100 mm in both thickness and height. The interior steel ring was designed with distinct thicknesses of 5 mm, 12 mm, and 25 mm, respectively. Meanwhile, a control group was included, which lacked an interior steel ring during the testing process. The circular restraint ring is visually represented in Figure 3.

**Figure 4:**
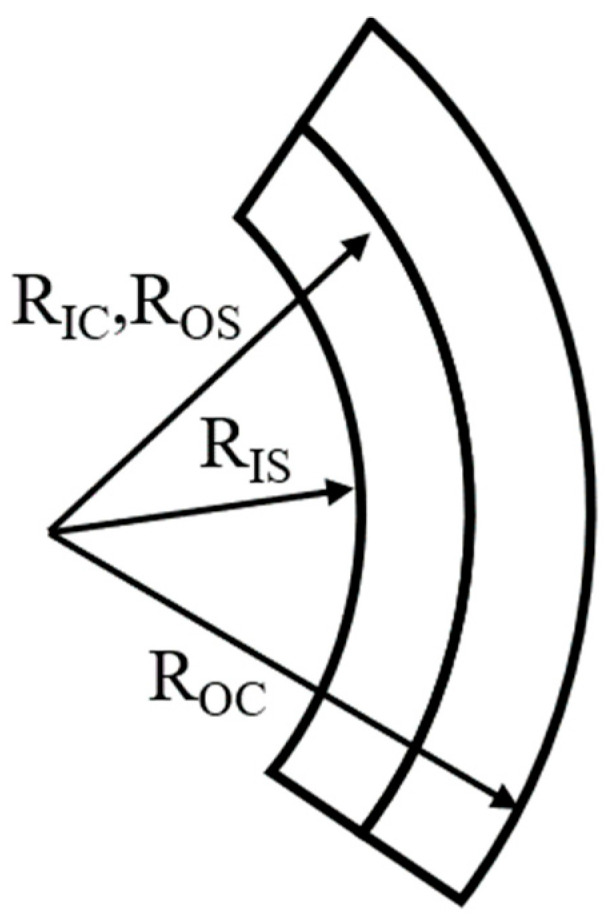
A diagram of the concrete restraint ring radius.

To determine the levels of restraint, the design relied on the application of Equation (1) [28]. The ultimate restraint degree for each test group is delineated in Figure 4 and Table 3.

(1) $$DR=\frac{1}{1+\frac{\left[\left(1+v_{S}\right)R_{IS}^{2}+\left(1-v_{S}\right)R_{OS}^{2}\right]\left[E_{C}\left(R_{OC}^{2}-R_{IC}^{2}\right)\right]}{\left[E_{S}\left(R_{OS}^{2}-R_{IS}^{2}\right)\right]\left[\left(1+v_{C}\right)R_{OC}^{2}+\left(1-v_{C}\right)R_{IC}^{2}\right]}}$$

DR = degree of restraint [%]. The parameter represents the proportion of concrete potential free volumetric shrinkage that is mechanically restricted by the external rigid structure (i.e., the steel ring).

$R_{OS},R_{IS}$ = Outer radius of the steel ring, interior radius of the steel ring;

$R_{OC},R_{IC}$ = Outer radius of the concrete ring, interior radius of the concrete ring;

$v_{S},v_{C}$ = Poisson’s ratio of the steel ring, taken as 0.3, and Poisson’s ratio of the concrete, taken as 0.2;

$E_{S},E_{C}$ = The elastic modulus of the steel ring, taken as 200 GPa, and the elastic modulus of concrete. The elastic modulus of concrete is known to vary over time. For the calculation, the value assigned to the 3-day elastic modulus of the concrete was 35.30 GPa.

#### 2.2.2. Shrinkage Deformation Test

Traditionally, strain gauges are the standard sensors used to assess the shrinkage of restrained concrete ring specimens. This approach has facilitated extensive research into how parameters including ring thickness [29], geometric configuration [30], environmental relative humidity [31,32] and control concrete shrinkage. However, since these sensors measure the deformation of the inner steel ring to indirectly calculate concrete strain, the resulting data is inherently biased by the material properties and dimensions of the steel mold [26]. Compounding this limitation, the well-documented issue of sensor data drift renders strain gauges highly unreliable for the long-term monitoring of strain evolution.

To bridge these methodological gaps, a novel experimental approach that integrates conventional strain gauges with DIC technology for investigating the early-age shrinkage of concrete rings under varying restraint levels is proposed in this study. In this dual-measurement setup, strain gauges indirectly capture the average internal stress–strain state of the concrete by measuring the deformation of the inner steel ring. Simultaneously, DIC directly quantifies both global and highly localized deformation fields on the exposed concrete surface. By coupling internal average measurements with external full-field surface mapping, this synergistic approach enables a comprehensive, multi-dimensional evaluation of the overall concrete strain behavior.

**Figure 5:**
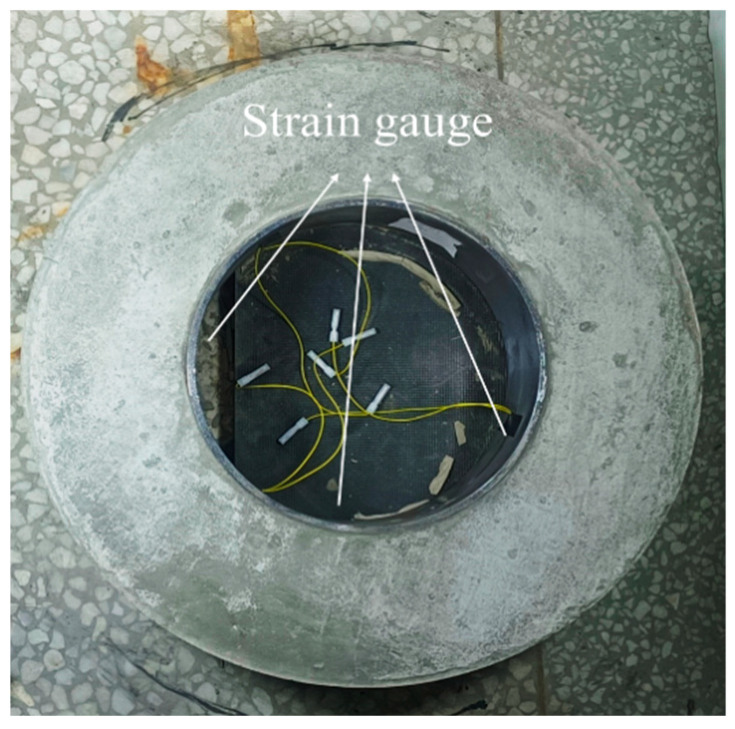
Mounting position of strain gauges.

For the internal strain measurement, three strain gauges were affixed to the inner surface of the steel ring at its mid-height, as illustrated in Figure 5. To ensure a representative and uniform strain profile, these gauges were spaced equidistantly along the circumference. All sensors were subsequently wired to a data acquisition system for continuous monitoring.

Surface strains of the concrete specimens were measured with foil-type electrical resistance strain gauges (Model 120-80AA), purchased from Xingdongfang Enterprise Store, Chengdu, Sichuan Province, China. To ensure representative measurements across the heterogeneous concrete matrix and accommodate the 20 mm nominal maximum aggregate size (NMAS), an 80 mm gauge in length was deliberately selected. The gauges consisted of a constantan foil grid on a glass-fiber-reinforced epoxy backing, featuring a nominal resistance of 120 ± 0.5 Ω and a gauge factor of 2.11 ± 1%. Prior to installation, the concrete surface was meticulously polished and cleaned. The gauges were bonded with a rapid-curing cyanoacrylate adhesive and subsequently sealed with a polyvinyl chloride (PVC) coating to prevent moisture ingress and mechanical damage. Strain data were continuously recorded via a JM3812 static strain data acquisition system (accuracy: ±0.2% F.S. ± 1 με). To eliminate thermally induced strain variations, temperature compensation was achieved by employing a dummy gauge affixed to an identical, unconstrained concrete control specimen situated in the same ambient environment.

**Figure 6:**
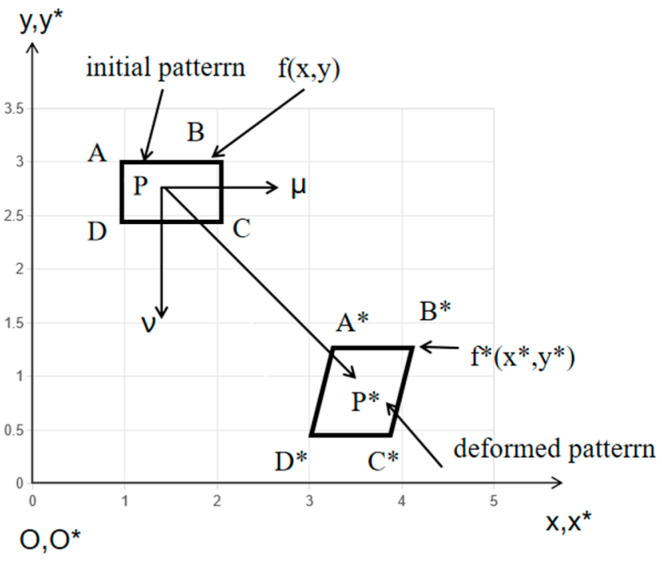
Schematic diagram of digital image correlation.

The DIC is an optical method for tracking displacements in images based on changes in the gray scale intensity of an applied speckle pattern [33]. A point in (x, y) in the undeformed (original) image is correlated with a point −(x*, −y*) in the deformed image as mentioned in Equation (2) and Figure 6. DIC-based virtual extensometer can measure the displacement at a designated point by tracking the relative motion of selected gauge points, providing a pointwise displacement from full-field data [34].

(2) $$\begin{array}{l} x*=x+\mu (x,y)\\ y*=y+\nu (x,y) \end{array}$$

The variables μ and ν represent the horizontal and vertical displacements, respectively.

**Figure 7:**
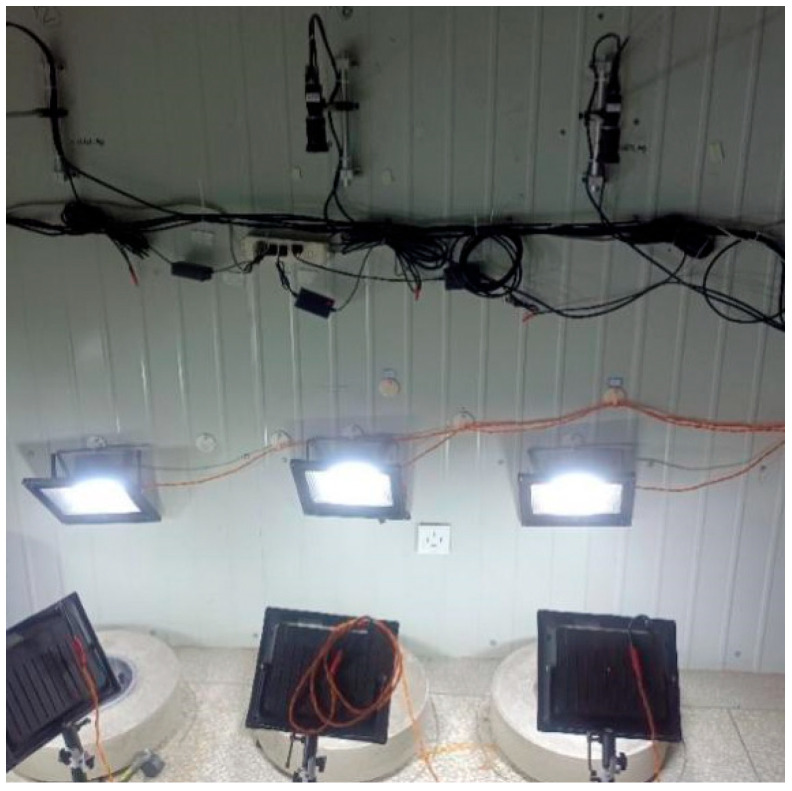
DIC test system.

DIC technology was used to quantify concrete shrinkage, as shown in Figure 7. Each experimental setup was equipped with a 12-megapixel industrial camera, purchased from Hikvision, Hangzhou, Zhejiang Province, China. accompanied by a pair of light sources. The specimen was placed on a rubber pad to minimize the effects of vibration while maintaining the bottom of the concrete ring in contact with the air. The industrial camera was mounted on the wall at a distance of 1000 mm from the surface of the specimen, with light sources symmetrically installed on either side to ensure ample and uniform lighting. Images were captured at a predetermined constant frequency by the camera, which was interfaced with a computing system via a data cable.

Unlike traditional strain collection instruments with fixed resolution, DIC technology offers a highly adaptable measurement resolution. This spatial precision is inherently variable, depending on the image dimensions, camera specifications, lens type, and the working distance. For example, when using a 12-megapixel camera (yielding a 4024 × 3036 pixel array), the ultimate physical resolution is governed by the sub-pixel accuracy of the tracking algorithm. If a 425 mm sample span corresponds to 3036 pixels in the captured image, and the algorithm achieves a sub-pixel accuracy of 0.2 pixels, the theoretical spatial resolution is calculated as (425/3036) × 0.2 = 0.028 mm. Consequently, researchers can seamlessly scale the measurement resolution to suit specific experimental requirements by simply adjusting the camera position or focal length. However, achieving this theoretical precision in practice relies heavily on the quality of the captured images, which necessitates rigorous control over camera parameters, lighting setups, and the ambient optical environment.

## 3. Results and Discussions

### 3.1. Restrained Shrinkage Analysis with Strain Gauge Method

To accurately quantify the early-age restrained shrinkage of concrete, strain gauges were installed at three equidistant locations along the inner periphery of the steel ring (Figure 5). It should be noted that this method is not applicable to the 0% restraint case; instead, strain gauges were only adopted for shrinkage evaluation of concrete rings under restraint levels of 35%, 55% and 75%.

**Figure 8:**
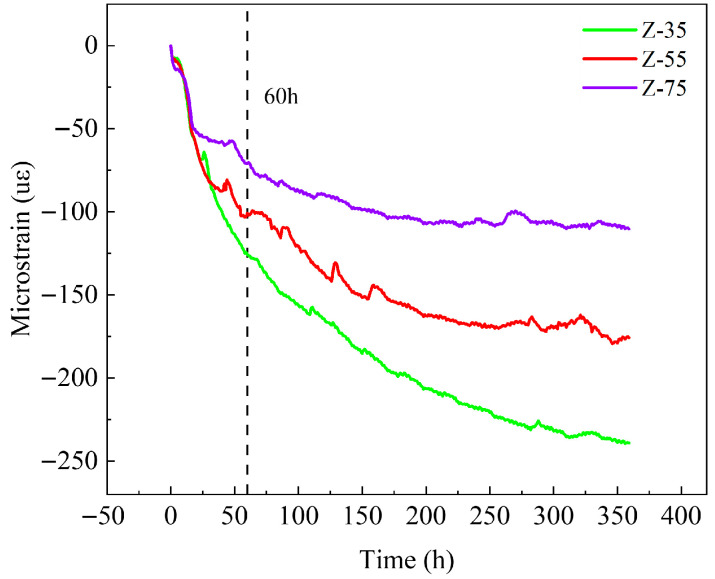
The strain of the inner steel ring under various degrees of restraint over time.

The temporal evolution of strain measured by the inner strain gauges under the different restraint levels is illustrated in Figure 8. While the strain development curves exhibit similar generalized trends over time regardless of the restraint degree, the magnitude of the strain is clearly inversely proportional to the level of restraint. Specifically, at any given moment, a higher degree of restraint consistently corresponds to a reduction in the measured strain—a finding that closely aligns with the observations reported by Yoo et al. [35].

According to the 15-day strain data listed in Table 4, specimens under 55% and 75% restraint levels exhibited strain decreases of 26.8% and 54.0%, respectively, compared to the 35% restraint level. A thicker inner steel ring possesses greater rigidity, which reduces its deformation under a given stress and exerts stronger restraint on the surrounding concrete ring [35]. This enhanced restraint generates higher restraint-induced stress within the concrete, leading to greater restrained shrinkage. Furthermore, the steep curves in Figure 7 indicate that concrete shrinkage increases rapidly within the first 60 h.

### 3.2. Restrained Shrinkage Analysis with DIC Method

Currently, DIC technology is utilized to observe local strain, settlement, and crack development on mortar surfaces [24,25,26,27], and to examine the impact of relative humidity and aggregate size on the non-uniform stress and drying cracks that develop in concrete following sectioning [36,37,38,39]. Limited research has applied DIC technology to directly observe concrete shrinkage strain [18], in which the impact of steel ring thickness on the degree of shrinkage in concrete was not considered. This study utilizes DIC to assess the impact of steel rings with varying thicknesses (0 mm, 5 mm, 12 mm, and 25 mm) on the shrinkage strain of concrete rings. The observation period lasted for 180 days.

Fundamentally, concrete shrinkage cracking is a mechanical failure process governed by restrained deformation. As the material undergoes volumetric contraction—driven by desiccation, hydration reactions, or thermal cooling—any restraint opposing this movement inevitably induces passive tensile strains within the cementitious matrix. With the continuous accumulation of shrinkage, once the induced maximum principal tensile strain exceeds the inherent ultimate tensile strain capacity of concrete, physical rupture of the internal microstructure occurs. This localized failure triggers the rapid propagation and coalescence of microcracks, ultimately manifesting as macroscopic shrinkage cracks on the surface. Consequently, the maximum principal strain (e1) can serve as a highly reliable indicator for identifying strain concentrations and zones of potential cracking risk.

**Figure 9:**
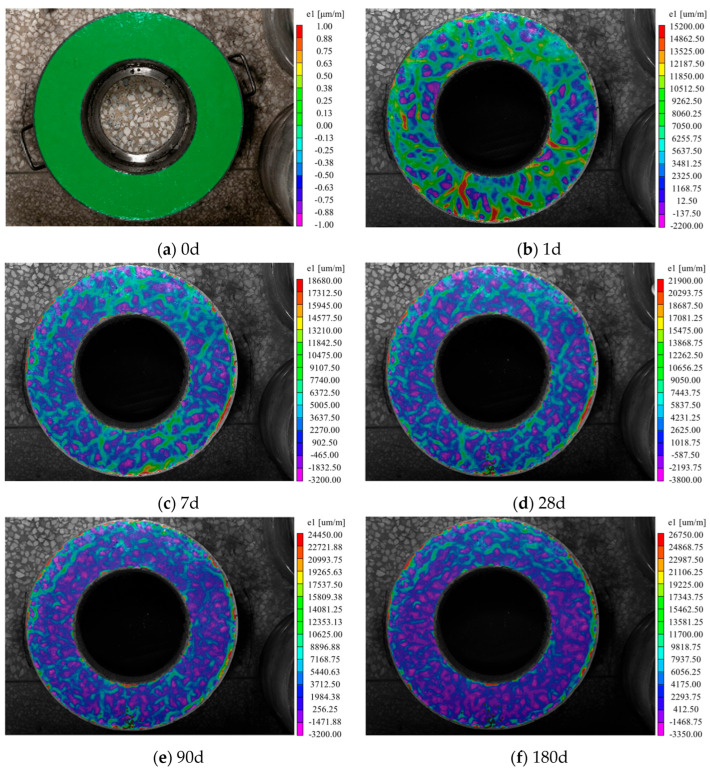
Contour map of maximum principal strain for Z-0.

**Figure 10:**
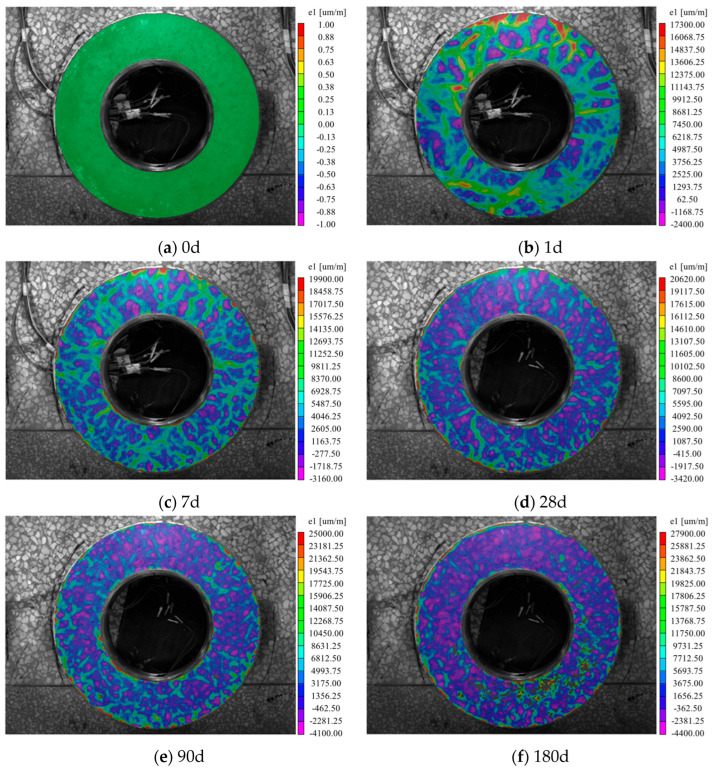
Contour map of maximum principal strain for Z-35.

**Figure 11:**
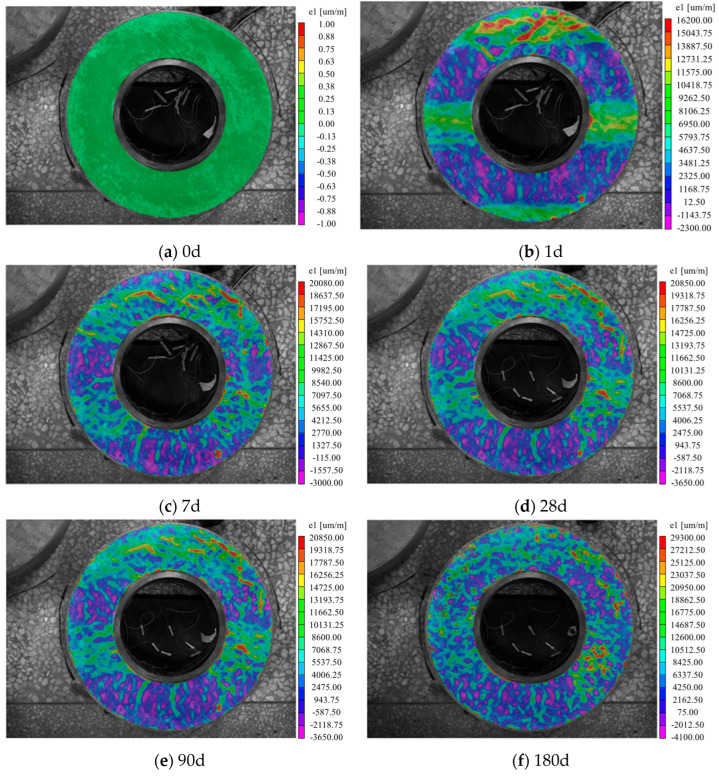
Contour map of maximum principal strain for Z-55.

**Figure 12:**
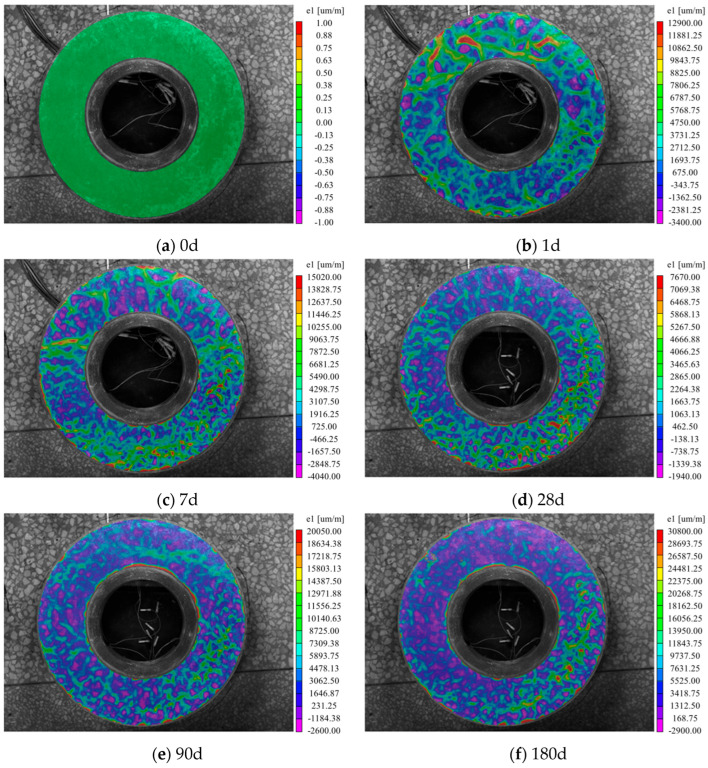
Contour map of maximum principal strain for Z-75.

The contour map variation in maximum principal strain e with age and restraint is drawn to monitor and assess the overall strain on the surface of the concrete ring shown in Figure 9, Figure 10, Figure 11 and Figure 12.

It can be seen in Figure 9, Figure 10, Figure 11 and Figure 12, for all restraint levels, both the maximum tensile strain and the area of tensile strain increase with age. The growth is most pronounced during the first day (0–1 d) with the maximum tensile strain primarily distributed along the inner and outer boundaries of the concrete ring. At any given age, higher restraint levels correspond to a greater maximum tensile strain. This corresponds to the primary cracking risk zones of the ring, aligning with previous studies that identify a high susceptibility to cracking between 7 and 14 days [11].

The implications of these findings are twofold. First, they verify the critical importance of curing during the initial seven days after placement [18], which is the optimal period for strength development. Second, concrete surfaces adjacent to the restrained side exhibit a heightened propensity for shrinkage and cracking, arising from the gradual weakening of the restraint force toward the unrestrained side. These findings further confirm that the first seven days represent the most necessary and effective window for curing freshly cast concrete. Since surfaces near the restraining boundary are highly prone to shrinkage and cracking, implementing proper pre- and post-conditioning measures is essential. Furthermore, the outermost concrete layer is subjected to moisture evaporation in three directions, which accelerates water loss and consequently reduces hydration.

**Figure 13:**
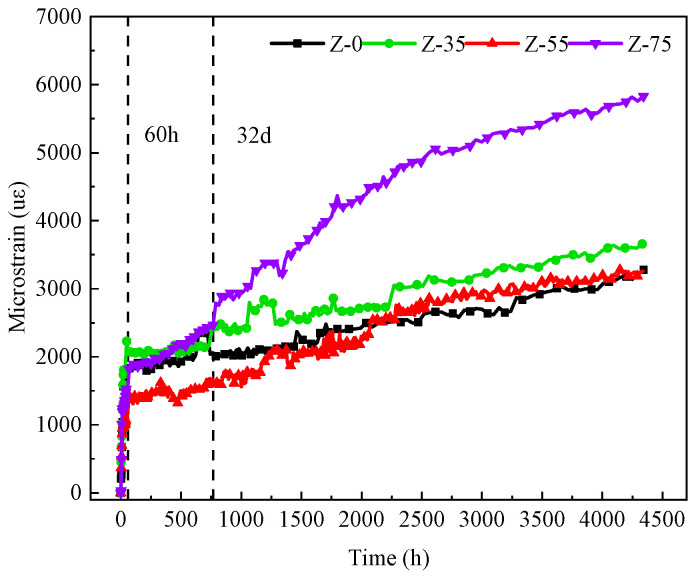
The average e1 variation in the concrete surface under different restraint degrees.

DIC technology is proven highly effective for evaluating surface shrinkage, providing accurate full-field deformation data during the entire curing and loading process. In contrast to indirect measurements acquired via embedded strain gauges, DIC facilitates the direct quantification of concrete shrinkage behavior. To facilitate a more intuitive analysis, the e1 strain contour data is plotted as a curve in Figure 13, in which it is presented that the evolution of average surface e1 strain with restraint degree, and the averaged strain characterizes the overall degree of surface deformation.

As shown in Figure 13, the average surface strain e1 increases over time following a consistent trend across all restraint levels. The total measured strain can be decomposed into three primary components: autogenous shrinkage (arising from cement hydration and pore structure changes), drying shrinkage (driven by moisture evaporation), and restraint-induced strain (imposed by the steel ring).

During the early hydration stage, drying shrinkage dominates the deformation development. The inner steel ring alters the moisture evaporation path. Without the steel ring, moisture evaporates from three exposed surfaces; by contrast, under restrained conditions, the drying path is limited to only two surfaces. Consequently, the unrestrained Z-0 sample exhibits larger initial drying shrinkage than the others. Among the restrained specimens, the excessive restraint of Z-75 renders restraint-induced strain the dominant shrinkage component, resulting in the maximum overall deformation. Conversely, Z-55 yields the lowest shrinkage at this stage. This low early-age shrinkage of Z-55 can be plausibly attributed to matrix densification induced by moderate restraint, a phenomenon that is not clearly observed in Z-35.

In the late hydration stage, concrete shrinkage is primarily governed by the restraint imposed by the steel ring. Higher restraint elevates the tensile stress in the concrete and accelerates the shrinkage rate. At this stage, Z-75 still exhibits the maximum shrinkage, whereas Z-0 shows the minimum value. The shrinkage of Z-55 gradually exceeds that of Z-0 while remaining lower than that of Z-35. Since the degree compaction of Z-55 remains lower than that of Z-35, this observation aligns with the previously discussed principle that the magnitude of constrained stress is directly proportional to the extent of annular deformation in the concrete. Finally, consistent with the inner strain gauge data, the first 60 h after casting constitutes the most critical period for concrete deformation. After the rapid deformation stage, the average strain e1 under all restraint levels remains relatively stable within the first month.

**Figure 14:**
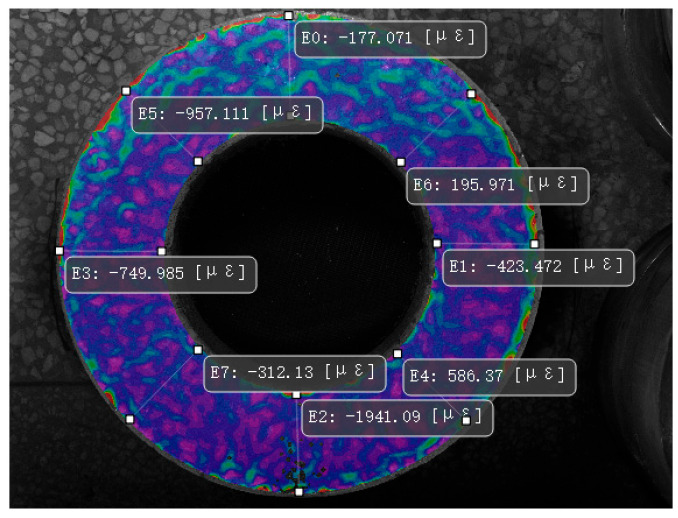
The arrangement of virtual extensometers on specimens.

The virtual extensometer is widely adopted in DIC application to assess shrinkage-induced cracking of concrete. This technique enables the precise measurement of the distance variation between two preselected points on a specimen over time. In this study, eight virtual extensometers were arranged at equal angular intervals around the circumference of concrete ring specimens under different restraint levels shown in Figure 14. The initial gauge length of each extensometer was set to 100 mm and calibrated according to the radial thickness of the concrete ring.

**Figure 15:**
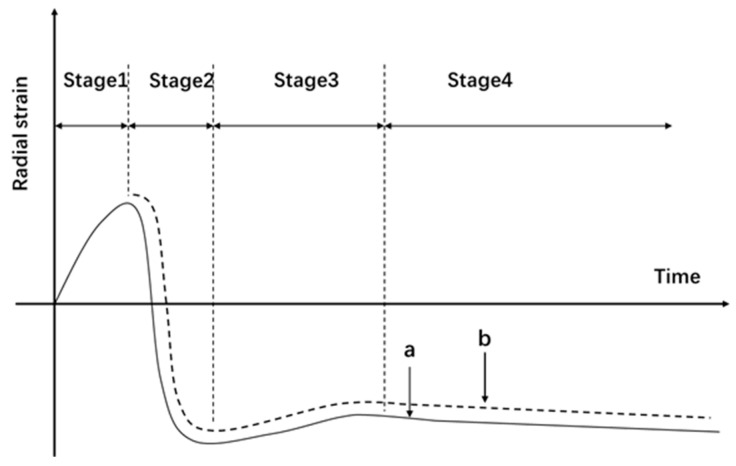
The stages of radial strain development in concrete rings.

Curve a in Figure 15 illustrates the shrinkage evolution of a conventional restrained ring test, which is divided into four distinct phases of strain evolution: initial fluctuations accompanied by slight elongation, a rapid shrinkage stage, a subsequent rebound to minor elongation and a final steady contraction stage. Specifically, the initial elongation (Stage 1) results from thermal expansion due to early-age hydration heat. As hydration accelerates, rapid autogenous and early drying shrinkage dominate (Stage 2). The subsequent strain rebound (Stage 3) is caused by stress relaxation following micro-crack initiation. Finally, steady contraction (Stage 4) occurs as moisture equilibrates, controlled by long-term drying shrinkage and creep. Each phase corresponds to specific physicochemical processes governing the shrinkage of the concrete ring: exothermic hydration reactions, thermal contraction, autogenous shrinkage and desiccation shrinkage [10,40,41]. However, the strain gauge, the e1 measurement system and the virtual extensometer did not record data during this initial phase in this study. As depicted by Curve b in Figure 15, the measured shrinkage initiates directly at Stage 2 for data acquisition commenced three hours after casting.

**Figure 16:**
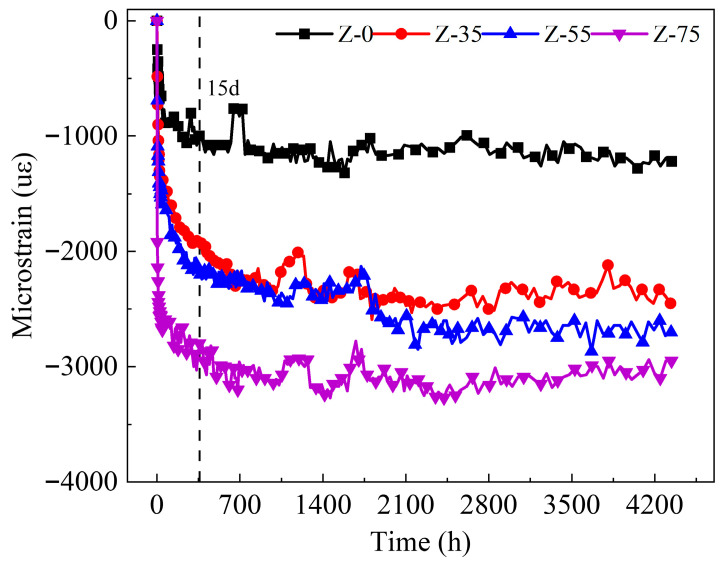
Radial displacements monitored by virtual strain gauges under varying degrees of restraint over a 180-day period.

It is presented that the temporal evolution of shrinkage strain acquired via DIC-based virtual extensometers with all plotted data corresponding to the averaged results from eight measuring points in Figure 16. Across all restraint levels, the restrained shrinkage consistently increases with age. The shrinkage strain under the 75% restraint level is the highest; correspondingly, as the degree of restraint decreases, the overall shrinkage of the concrete ring also diminishes.

The strain gauges measure the strain at the midpoint of the inner steel ring. A higher restraint level, corresponding to a larger steel ring thickness, provides greater resistance to the inward radial pressure induced by concrete shrinkage. Consequently, the measured compressive strain in the steel ring decreases for a given concrete mixture. DIC measurements reveal that the surface deformation of the concrete is primarily driven by surface moisture loss, while also being affected by tensile stresses induced by external restraint. Under greater restraint, the concrete experiences higher tensile stress, which may alter the pore structure by increasing porosity and pore size. This potential modification could accelerate moisture evaporation and further intensify shrinkage. Consequently, the recorded surface shrinkage strains increase proportionally with the degree of restraint.

The strain measured by the virtual extensometer is shown in Table 5.

As presented in Figure 16 and Table 5, the radial strain of the concrete measured by the virtual extensometers consistently increases with curing age. Higher restraint magnitude is positively correlated with greater shrinkage strain, while the difference between Z-35 and Z-55 specimens remains insignificant at early ages. By the 15th day, specimens under restraint levels of 35%, 55% and 75% exhibit strain increases of 76.7%, 105.8% and 196.1%, respectively, compared with the unrestrained reference group. The measured surface strain consists of three distinct components: (i) restraint-induced micro-strain on the concrete ring surface; (ii) autogenous shrinkage resulting from cement hydration and associated pore-structure changes under restraint; and (iii) drying shrinkage driven by moisture evaporation.

The discrepancy between the DIC and strain gauge measurements reflects a spatial gradient rather than a data contradiction. Strain gauges capture the volumetrically averaged internal deformation, which is directly restrained by the steel ring. In contrast, DIC measures localized surface shrinkage, which is driven primarily by intense moisture evaporation. The strain gauges measure the strain at the mid-height of the inner steel ring; therefore, as the steel ring becomes thicker, its ability to resist the shrinkage forces exerted by the concrete increases, resulting in a smaller strain recorded within the steel ring itself. In contrast, the DIC method captures the deformation directly on the concrete surface. This surface deformation is primarily driven by moisture loss, yet it remains coupled to the overall restraint forces acting on the concrete ring. Consequently, a higher degree of restraint induces greater internal stress, which ultimately manifests as a larger measured shrinkage strain on the concrete surface.

Table 5 further demonstrates that shrinkage occurs predominantly at an early age. Under the 75% restraint level, surface shrinkage within the first three days constitutes 78.4% of the total shrinkage, a finding that corroborates the internal strain gauge observations. Consequently, the critical cracking-risk window for the concrete rings is concentrated within the first week post-casting, aligning with previous studies that report a heightened cracking propensity between 7 and 14 days [11].

Under the 75% restraint level, the 15-day surface shrinkage reaches 2750 με, far exceeding the 110.33 με recorded by the internal strain gauges. This substantial discrepancy between the surface and internal measurements stems from the combined effects of drying and restrained shrinkage. Because the concrete surface is directly exposed to the external environment, it experiences active heat and moisture exchange. Consequently, surface-drying shrinkage governs the localized behavior, causing a rapid rise in capillary pressure that generates significantly larger shrinkage strains [42]. This severe moisture loss concurrently reduces the local degree of hydration and lowers the elastic modulus, rendering the surface material more susceptible to deformation under restraint. Furthermore, excessive confinement coarsens the overall pore structure of the concrete, which accelerates moisture egress, exacerbates drying, and markedly increases the shrinkage rate. In contrast, the strain gauges located at the mid-height of the inner steel ring capture only the average internal strain of the bulk concrete. This fundamental difference in measurement scope—localized surface extremes versus internal volumetric averages—explains why the DIC method consistently records substantially greater shrinkage than the conventional strain gauges.

Early-age, near-surface cracking induced by concrete shrinkage is typically more severe than that within the internal core. This phenomenon is primarily driven by the combined effects of humidity gradients, thermal variations, and moisture evaporation. The initiation and propagation of these surface cracks severely compromise both structural durability and interfacial bonding. Consequently, this non-uniform distribution of shrinkage magnifies the threat to the integrity of the concrete cover and its long-term performance, making it a critical consideration for both structural design and construction. This aligns with the assertion by Bažant et al. [43] that the maintenance of concrete structures fundamentally begins with the observation of surface cracks and degradation.

Consequently, mitigation strategies must target surface shrinkage independently, rather than relying solely on bulk volume control. Focusing exclusively on overall deformation risks overlooking the early initiation and propagation of surface cracks, which ultimately compromises both aesthetics and durability. Recognizing this dominant role, localized early surface-shrinkage management must be integrated into core design frameworks. This comprehensive strategy encompasses optimized material selection, protective cover design, interfacial treatments, specific curing regimes, and structural layouts, alongside active humidity maintenance to restrict moisture evaporation. Ultimately, these targeted measures protect internal reinforcement, mitigate early-age corrosion risks, and ensure long-term structural durability.

It should be noted that the present conclusions were validated under controlled indoor environments using specific mix proportions. In practice, scale effects, combined with complex in situ variables such as temperature–humidity fluctuations, structural loading, and wind-induced drying, may significantly alter the magnitude and evolution of the shrinkage gradient. Therefore, future research should prioritize scaled-up structural testing, long-term field monitoring, and investigations into how chemical admixtures influence this surface-to-core gradient. Finally, integrating these empirical field observations into a coupled physico-numerical model will be essential for enhancing the predictive accuracy of concrete shrinkage behavior.

## 4. Conclusions

To investigate the effect of varying restraint levels (0%, 35%, 55%, and 75%) on concrete shrinkage, this study integrated digital image correlation (DIC) for surface strain mapping with traditional strain gauges for internal steel ring measurements. The principal conclusions are as follows:

1. Internal strain gauge measurements indicate that the recorded compressive shrinkage strain of the inner steel ring decreases with an increase in restraint level. Furthermore, this deformation is mainly concentrated during the early-age hydration phase.
2. DIC surface measurements demonstrate that the localized concrete shrinkage strain increases proportionally with restraint level, and this phenomenon predominantly occurs at early ages. Furthermore, the application of DIC proved highly effective for characterizing this behavior. By providing reliable, full-field surface deformation data throughout the curing and loading phases, DIC enables the direct quantification of concrete shrinkage evolution—offering a significant analytical advantage over the indirect data yielded by conventional internal strain gauges.
3. Cross-validation between the strain gauge and DIC measurements reveals that, influenced by factors such as moisture variations, the specimen surface exhibits a significantly more pronounced deformation tendency than its interior. Notably, a non-linear phenomenon was observed: the average principal strain (e1) obtained directly from the DIC contour maps reached its minimum magnitude at the 55% restraint level. It should be explicitly clarified that this minimum applies solely to the average e1 field values and must not be interpreted as a general conclusion for overall surface shrinkage, given that the radial strains determined via the virtual extensometers demonstrate a distinctly different trend (i.e., continuously increasing with higher restraint). Nevertheless, this specific finding suggests that when evaluating the risk of shrinkage cracking, a lower degree of restraint does not invariably guarantee greater safety. Instead, local structural stiffness matching and deformation compatibility exert a complex influence on the distribution of the surface strain field.

The surface concrete experiences significantly greater shrinkage than the interior. Post-pour curing is necessary, and the surface layer should adopt an independent shrinkage-control design strategy rather than relying solely on overall shrinkage control. These findings are based on the specific concrete mix, specimen geometry, and controlled laboratory conditions used in this study. Further research involving larger-scale elements and varying field environments is necessary to generalize these observations.

## Figures and Tables

**Table 1:** Concrete mix proportions (kg/m^3^).

Water	Cement	Fly Ash	Fine Aggregate	Coarse Aggregate
153	310	130	745	1045

**Table 2:** Physicochemical properties of cement and fly ash.

Parameter	Cement (%)	Fly Ash (%)
SiO_2_	22.05	65.59
Al_2_O_3_	4.95	23.37
Fe_2_O_3_	3.55	4.33
Na_2_O	0.40	1.54
MgO	1.45	1.36
K_2_O	0.50	1.02
CaO	65.40	2.28
MnO	0.27	-
SO_3_	1.42	0.50
Specific gravity (kg/m^3^)	3100	2450
Specific surface area (m^2^/kg)	385.5	310.0

**Table 3:** The degree of restraint for each test group.

Thickness of Interior Steel Ring/mm	0	5	12	25
Degree of restraint	0	35%	55%	75%
Test group	Z-0	Z-35	Z-55	Z-75

**Table 4:** Final steel ring strains under different restraint levels at 15 days.

Group	Z-35	Z-55	Z-75
Microstrain	−239.04	−175.64	−110.33

**Table 5:** Surface radial strains of the concrete extracted using virtual strain gauges.

Group	Z-0	Z-35	Z-55	Z-75
Microstrain of 3 d/uε	−780	−1140	−1670	−2590
Microstrain of 7 d/uε	−870	−1430	−1960	−2750
Microstrain of 15 d/uε	−996	−1760	−2050	−2950
Microstrain of 90 d/uε	−1091	−2430	−2670	−3130
Microstrain of 180 d/uε	−1220	−2450	−2700	−3300

## Data Availability

The data presented in this study are available on request from the corresponding author as the results are for use in nuclear power plants and must be strictly confidential.
